# Validation of a multicolor staining to monitor _phospho_STAT5 levels in regulatory T-cell subsets

**DOI:** 10.18632/oncotarget.6486

**Published:** 2015-12-07

**Authors:** Grégory Ehx, Muriel Hannon, Yves Beguin, Stéphanie Humblet-Baron, Frédéric Baron

**Affiliations:** ^1^ Hematology Research Unit, Groupe Interdisciplinaire de Génoprotéomique Appliquée (GIGA)-I^3^, University of Liège, Liège, Belgium; ^2^ Department of Medicine, Division of Hematology, CHU of Liège, Liège, Belgium; ^3^ Autoimmune Genetics Laboratory, University of Leuven, Leuven, Belgium

**Keywords:** Treg, regulatory T cells, FOXP3, STAT5, IL-2, Immunology and Microbiology Section, Immune response, Immunity

## Abstract

**BACKGROUND:**

Regulatory T cells (T_regs_) are key players in immune tolerance. They express the transcription factor FOXP3 and are dependent of the STAT5 signaling for their homeostasis. So far, the study of phosphorylated epitopes by flow cytometry required treating the cells with methanol, which is harmful for several epitopes.

**METHODS:**

Here we assessed whether the PerFix EXPOSE reagent kit (PFE)(Beckman Coulter) allowed monitoring the phosphorylation level of STAT5 in T_reg_ subpopulations together with complex immunophenotyping. Results observed with the PFE kit were compared to those observed without cell permeabilization for surface markers, with paraformaldehyde permeabilization for non-phosphorylated intracellular epitopes, and with methanol-based permeabilization for _phospho_STAT5 staining.

**RESULTS:**

In human PBMCs, the PFE kit allowed the detection of surface antigens, FOXP3, KI67 and _phospho_STAT5 in similar proportions to what was observed without permeabilization (for surface antigens), or with PFA or methanol permeabilizations for FOXP3/KI67 and _phospho_STAT5, respectively. Comparable observations were made with murine splenocytes. Further, the PFE kit allowed determining the response of different human and murine T_reg_ subsets to IL-2. It also allowed demonstrating that human T_reg_ subsets with the highest levels of _phospho_STAT5 had also the highest suppressive activity *in vitro*, and that anti-thymocyte glogulin (ATG) induced T_reg_ independently of the STAT5 pathway, both *in vitro* and *in vivo*.

**CONCLUSIONS:**

We have validated a multicolor staining method that allows monitoring _phospho_STAT5 levels in T_reg_ subsets. This staining could be useful to monitor responses of various T_reg_ subsets to IL-2 therapy.

## INTRODUCTION

Regulatory T cells (T_regs_) represent a fraction of CD4^+^ T cells that are indispensable for maintaining immunological self tolerance [1, 2]. They express the forkhead box protein 3 factor (FOXP3) in their nuclei, and CD25 (the high-affinity component of the trimeric form of the interleukin 2 (IL-2) receptor) on their cell surface [3, 4]. T_regs_ are also featured by a low expression of CD127, the α chain of the IL-7 receptor [5].

The main population of T_regs_ is generated in the thymus, where high-avidity recognition of self-antigens by their T-cell receptor (TCR) leads to the generation of thymus T_regs_ [6, 7]. However, T_regs_ can also be generated in the periphery (peripheral T_regs_) from conversion from conventional CD4^+^ T cells (T_convs_), or *in vitro* from naive T_convs_ by TCR stimulation along with TGF-β, IL-10 or retinoic acid signaling (induced T_regs_) [6]. In addition to distinctions based on their origin, T_reg_ can be further subdivided into central or naive T_regs_ (that are CD45RA^+^CCR7^+^ in humans and CD62L^hi^CCR7^+^ in mice), and effector T_regs_ (that are CD45RA^neg^ in humans, and CD62L^low^CCR7^low^CD44^high^ and CD103^+^ in mice) [4, 7–9]. Importantly, in humans, effector T_regs_ can be further separated between activated (HLA-DR^+^) effector T_regs_ that are highly proliferating, and HLA-DR^neg^ effector T_regs_ that are less proliferating [9].

In the last decade, animal studies have evidenced that restoring the T-cell balance in favor of T_regs_ allowed the control of autoimmunity in several animal models of rheumatologic diseases [10]. Further, T_reg_ administration prevented graft-*versus*-host disease (GVHD, a redoubtable complication of allogeneic hematopoietic cell transplantation caused by donor immune cells contained in the graft reacting against recipient healthy tissues [11]) both in mouse to mouse and in humanized mouse models of GVHD [12–15]. Further, T_reg_ infusion also prolonged human skin graft survival in humanized mice [16].

These observations in animal models prompted the initiation of pilot clinical studies of T_reg_ infusion as prevention or treatment of GVHD [17–19], or as prevention of solid organ rejection [20].

IL-2 is a member of the common cytokine gamma chain family that plays a central role in T_reg_ homeostasis through stimulation of the STAT5 pathway [4, 21]. This prompted Koreth *et al.* to investigate the safety and efficacy of low-dose IL-2 administration (with the aim of boosting T_regs_) in patients with chronic GVHD [22]. The authors observed that administration of low-dose IL-2 not only successfully increased T_reg_ blood counts but also induced clinical responses in half of the patients. Administration of low-dose IL-2 resulted also in increased T_reg_ counts and clinical responses in patients with autoimmune diseases such as hepatitis C virus-induced vasculitis [23] or type 1 diabetes [24]. With the development of such cytokine-based immunotherapies, monitoring of the phosphorylation level of key players in target signaling pathways (and particularly of STAT5), simultaneously in several cell sub-populations, is of great interest in order to assess treatment efficacy early.

So far, the study of phosphorylated epitopes by flow cytometry required treating the cells with methanol, which is harmful for many extra- and intra-cellular epitopes and compromises multiparameter analyses. Recently, a new reagent kit, the PerFix EXPOSE kit (Beckman Coulter), was designed to allow studying phosphorylated epitopes without compromising other epitopes. In the present report, we compared this new procedure with reference permeabilization protocols for (non)-phosphorylated epitopes to validate it and used it to study T_reg_ subsets response to IL-2 in human and mouse samples. Our results showed that the PerFix technique is suitable for combined _phospho_STAT5 monitoring and accurate immunophenotyping in human and mouse samples. We also highlighted differential responses to IL-2 among T_reg_ subsets.

## RESULTS AND DISCUSSION

### Validation of a multicolor staining to monitor _phospho_STAT5 levels in human T_reg_ subsets

To assess the capacity of the PFE kit to allow the accurate quantification of _phospho_STAT5 in combination with surface (CD4, CD25, CD127, HLA-DR and CD45RA) and non-phosphorylated intracellular (FOXP3, KI67) epitopes, we compared this procedure with the conventional permeabilization method for phospho-epitopes (Methanol (MeOH)-based method) and the conventional permeabilization procedure for FOXP3 and KI67 staining (Paraformaldehyde (PFA) -based method). In order to assess the impact of any permeabilization treatment on the expression of surface epitopes, cells were also analyzed after staining of surface epitopes without any further permeabilization. These comparisons were repeated twice with 8 healthy volunteers and similar results were found in each experiment. Results from the first experiment are presented hereafter as representative example. The following combination of antibodies was used: CD4-PE-Cy5, CD25-BV421, CD127-biotine-strepatavidine-PE-Cy7, CD45RA-BV510, HLA-DR-APC-efluor780, FOXP3-AlexaFluor488, KI67-PE and _phospho_STAT5-AlexaFluor647 (detailed in materials and methods).

**Figure 1:**
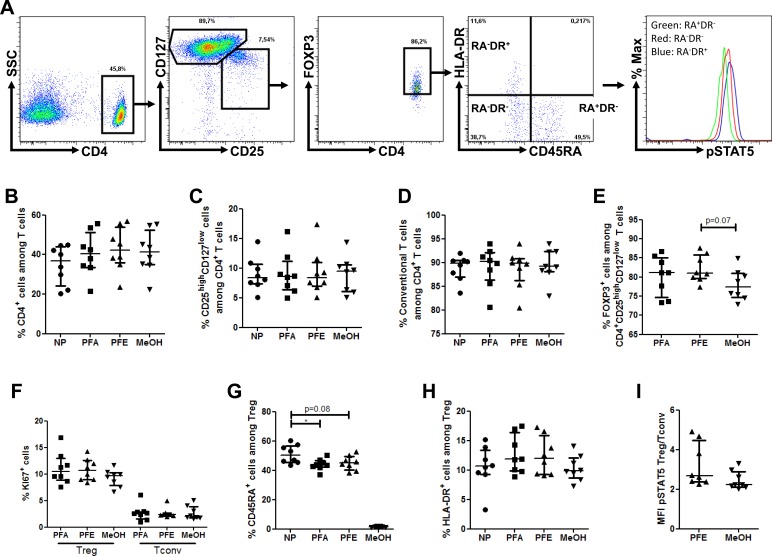
PerFix EXPOSE allows accurate detection of _**phospho**_STAT5 together with surface and intracellular immunophenotyping PBMC from 8 healthy volunteers were surface stained with anti-CD4, -CD25, -CD127, -CD45RA and -HLA-DR antibodies and were either non permeabilized (NP) or stained with anti-FOXP3, -KI67 and -_phospho_STAT5 antibodies after permeabilization with either paraformaldehyde (PFA)-based technique, PerFix EXPOSE (PFE) or methanol (MeOH)-based technique. Data show median values of 8 biological replicates / condition with interquartile range (* *p* < 0.05, **p < 0.005, ***p < 0.0005). **A.** Gating strategy of tubes permeabilized with the PerFix method. **B.-H.** Comparison of cell frequencies between the different permeabilization methods: CD4^+^ T-cell frequency among the total lymphocyte population **B.**, CD25^high^CD127^low^ among CD4^+^ T cells **C.**, T_conv_ (CD25^low/int^CD127^high^) among CD4^+^ T cells **D.**, FOXP3^+^ among CD25^high^CD127^low^ cells **E.**, KI67^+^ cell frequency among either T_reg_ or T_conv_ populations **F.**, CD45RA^+^ among CD4^+^CD25^high^CD127^low^FOXP3^+^ T_reg_ **G.** and HLA-DR^+^ among CD4^+^CD25^high^CD127^low^FOXP3^+^ T_regs_ **H. I.** Comparison of ratio, for each sample, of _phospho_STAT5 MFI of T_regs_ *versus*_phospho_STAT5 MFI of T_convs_ in PerFix and methanol-based methods.

Using the gating strategy described in Figure 1A, we observed similar frequencies of CD4^+^, CD25^high^CD127^low^ and CD25^low/int^CD127^high^ cells among non-permeabilized cells, and cells permeabilized with either the PFE, PFA or MeOH methods (Figure 1B–1D).

Next we compared FOXP3^+^ frequency within the CD25^high^CD127^low^ population and observed comparable FOXP3^+^ frequency among cells permeabilized with the PFA or with the PFE method, highlighting the successful and accurate staining of FOXP3 with this later procedure (Figure 1E). In contrast, there was a trend for decreased FOXP3^+^ frequency within the CD4^+^CD25^high^CD127^low^ population in cells permeabilized with the MeOH procedure. Regarding KI67 staining, no significant difference of frequency within either T_reg_ or T_conv_ populations was found between the three permeabilization protocols (Figure 1F).

Importantly, similar proportions of naive and activated effector T_regs_ were observed with the PFA and PFE procedures (Figure 1G–1H). In contrast, the methanol permeabilization procedure strikingly decreased the frequency of naive T_regs_, highlighting the deleterious impact of this permeabilization technique on certain surface epitopes such as CD45RA, although we can not exclude that by trying several CD45RA epitopes satisfying results could have been be achieved also with the methanol technique.

Next, we compared STAT5 phosphorylation levels (evaluated as mean fluorescence intensity (MFI) of _phospho_STAT5-APC) in T_regs_, normalized on T_conv phospho_STAT5 levels to reduce inter-assay variability of MFI between PFE and methanol permeabilization methods, after stimulation with 10 IU/ml of IL-2. Importantly, no difference between the two methods was observed (Figure 1I), demonstrating the reliability of the PFE technique for _phospho_STAT5 analysis. This experiment further evidenced higher levels of _phospho_STAT5 in T_regs_ than in T_convs_ following stimulation with low-dose IL-2, as recently observed by another group of investigators [24].

Finally, we assessed the variability for each donor between the PFE and reference technique (PFA or MeOH). Using simple linear regressions, we found a strong positive correlation (Spearman r ≥ 0.88, p ≤ 0.0072) between the results of the PFE and reference method for CD4^+^, CD25^high^CD127^low^, CD25^low/int^CD127^high^ (T_conv_), CD45RA^+^ T_reg_, HLA-DR^+^ T_reg_ and T_reg_/T_conv phospho_STAT5 ratio (Supplemental Figure 1). In contrast, there was no correlation for FOXP3^+^ and KI67^+^ cells frequency within CD25^high^CD127^low^ and T_regs_, respectively, likely because of the low inter-donor variability of these parameters. However, importantly, there was a significant correlation for the frequency of CD25^high^CD127^low^FOXP3^+^ cells (T_regs_) among total lymphocytes (*r* = 0.83, *p* = 0.0184) or CD4^+^ T cells (*r* = 0.97, *p* = 0.0004), as well as for the frequencies of the different T_reg_ subpopulations among CD4^+^ T cells (naive Treg: *r* = 0.88, *p* = 0.0072; effector Treg: *r* = 0.97, *p* = 0.0004; and activated effector Treg: *r* = 0.76, *p* = 0.0368).

Altogether these data validate an 8-color staining method allowing measurement of _phospho_STAT5 levels in human T_reg_ subsets.

### In human PBMCs, activated effector T_regs_ have higher _phospho_STAT5 levels than other T_reg_ subsets after stimulation with IL-2

Despite the growing knowledge on human T_regs_, little is known about the homeostatic properties of their sub-populations. Therefore, we took advantage of the PFE kit to study the respective capacities of the different T_reg_ subsets at phosphorylating STAT5 in response to IL-2.

**Figure 2:**
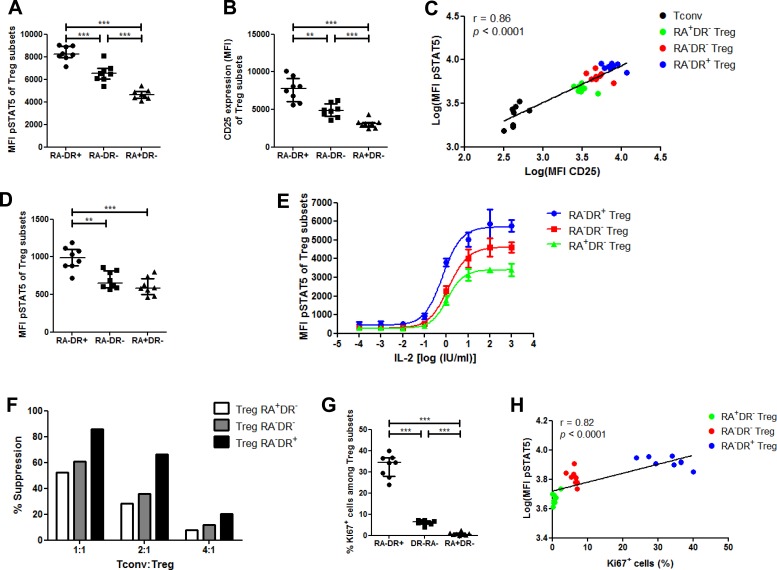
Phospho-STAT5 comparison between human T_**reg**_ subsets **A.-C.**, **G.-H.** PBMC from 8 healthy volunteers were stimulated with 10 IU/ml of recombinant human IL-2, stained with anti-CD4, -CD25, -CD127, -CD45RA and -HLA-DR antibodies, permeabilized with the PerFix EXPOSE method and stained with anti-FoxP3, -KI67 and -_phospho_STAT5 antibodies. **A.** Comparison of STAT5 phosphorylation level (MFI) between the different T_reg_ subsets with IL-2 stimulation, based on their expression of CD45RA and HLA-DR. **B.** CD25 expression (MFI) of the different T_reg_ subsets. **C.** Correlation between STAT5 phosphorylation level (log of MFI) and CD25 expression (log of MFI) in T_convs_ and the different T_reg_ subsets. **D.** Comparison of _phospho_STAT5 MFI between the different T_reg_ subsets without IL-2 stimulation. **E.** Total T cells were isolated from 3 healthy volunteers, stimulated with various concentrations of IL-2 for 15 min and _phospho_STAT5 level was measured in the different T_reg_ subsets after PerFix EXPOSE permeabilization. Data show mean values with standard deviation. **F.** Comparison of *in vitro* suppressive activity between the different human T_reg_ subsets at different T_reg_:T_conv_ ratios. Data show mean value of triplicates. **G.** Comparison of KI67^+^ cell frequency among the different T_reg_ subsets. **H.** Correlation between STAT5 phosphorylation level after stimulation with 10 IU/ml of IL-2 (log of MFI) and KI67^+^ cell frequency in the different T_reg_ subsets. Data show median values with interquartile range (* *p* < 0.05, **p < 0.005, ***p < 0.0005).

First we performed this comparison with low-dose (10 IU/ml) IL-2. The highest _phospho_STAT5 levels were found in activated effector T_regs_, followed by HLA-DR^neg^ effector T_regs_, while naive T_regs_ had the lowest levels of _phospho_STAT5 (Figure 2A). We then examined whether _phospho_STAT5 levels correlated with CD25 expression by T_regs_. We first observed that, as seen with _phospho_STAT5 levels, activated effector T_regs_ had the highest expression of CD25, followed by HLA-DR^neg^ effector T_reg_ and naive T_reg_ (Figure 2B). These data are in concordance with those reported by Miyara *et al.* showing higher CD25 expression in activated effector T_regs_ than in naive T_regs_ [8]. Further, taking data from T_conv_ and T_reg_ subsets together, there was a strong correlation between _phospho_STAT5 and CD25 levels (Spearman *r* = 0.86, *p* < 0.0001, Figure 2C). These findings demonstrate that the different T_reg_ subsets are not homogeneous in terms of response to IL-2 stimulation.

In order to verify that these observations remained present in basal conditions, we performed the same analyses with unstimulated T_regs_ and observed significantly higher _phospho_STAT5 levels in activated effector T_regs_ than in the two other T_reg_ subpopulations, while no differences were detected between HLA-DR^neg^ effector T_regs_ and naive T_regs_ (Figure 2D).

To further evaluate the response of the various T_reg_ subsets to IL-2, we compared _phospho_STAT5 levels in the three T_reg_ subsets after stimulation with an 8-log range of IL-2 concentration (Figure 2E and Supplemental Figure 2). As observed above, activated effector T_regs_ had the highest _phospho_STAT5 levels, followed by HLA-DR^neg^ effector T_regs_ and naive T_regs_. Specifically, the log half maximal effective concentration (log(EC_50_)) of activated effector T_regs_ (−0.165) was lower than the log(EC_50_) of HLA-DR^neg^ effector T_regs_ (0.096, *p* = 0.0495) and naive T_regs_ (0.039, *p* = 0.0768) subsets, supporting a higher proliferative capacity of this T_reg_ subset over the two others. These results are consistent with the differences observed in unstimualted T_regs_ and further demonstrate the heterogeneity of T_reg_ subsets in terms of response to IL-2 stimulation. In addition, our observations are in line with a recent study reporting that high-dose IL-2 therapy in melanoma patients promoted a T_reg_ subset with an activated phenotype [25]. Interestingly, we observed that the activated effector T_regs_ had the highest suppressive activity *in vitro,* while the two other populations presented similar (lower) suppressive activities (Figure 2F). These results suggest that _phospho_STAT5 level of T_reg_ subsets might be predictive of their suppressive function.

Finally, we compared KI67 expression between T_reg_ subsets and found the highest frequency of KI67^+^ cells within the activated effector T_reg_ fraction, while the HLA-DR^neg^ effector T_regs_ had a higher KI67 expression than naive T_regs_ (Figure 2G), in agreement with previous findings [9]. Given the pivotal function of STAT5 phosphorylation in T_reg_ proliferation, a significant correlation was found between KI67 expression of T_reg_ subsets and their level of _phospho_STAT5 after low-dose IL-2 stimulation (Spearman *r* = 0.82, *p* < 0.0001, Figure 2H).

Because cryopreserved rather than fresh PBMCs are frequently used in clinical trials, we assessed _phospho_STAT5 levels in T_reg_ subsets recovered from human cryopreserved PBMCs. As observed with freshly isolated PBMCs, activated effector T_regs_ from cryopreserved PBMCs presented the highest levels of _phospho_STAT5, CD25 and KI67 in comparison with the two other subsets, while effector T_regs_ had significantly higher levels of these markers than naive T_regs_ (Supplemental Figure 3). These data indicate that the PFE technique is reliable also for thawed PBMC samples.

Altogether these data suggest that the different proliferative capacities of T_reg_ subsets may be dependent upon their divergent capacities to respond to IL-2 through the STAT5 pathway.

### Impact of anti-thymocyte globulin (ATG) and cyclosporin A on T_reg phospho_STAT5 levels

**Figure 3:**
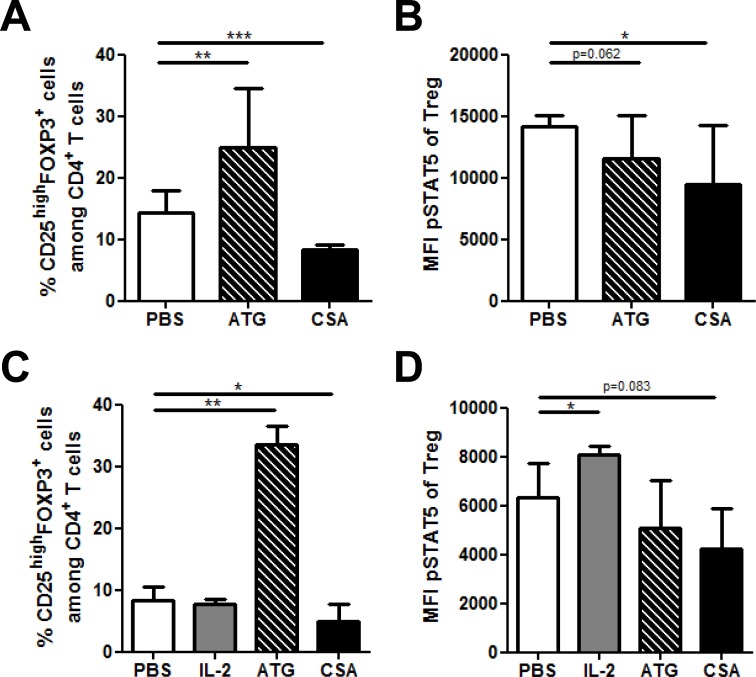
Impact of Anti-thymocyte globulin (ATG) and cyclosporin A (CSA) on T_reg phospho_STAT5 levels *in vivo* and *in vitro* **A.-B.** Human PBMCs, freshly isolated from 3 healthy donors were cultured in triplicate in the presence of either PBS, CSA (500 ng/ml) or ATG (100 μg/ml) for 24 hours. The impact of these compounds on CD4^+^CD25^high^FOXP3^+^ cell frequency **A.** as well as on _phospho_STAT5 levels in T_regs_ **B.** was assessed by flow cytometry using the PFE technique for permeabilization. Data show median values of 9 replicates (3 biological replicates in triplicates / condition) with interquartile range (* *p* < 0.05, **p < 0.005, ***p < 0.0005). (C-D) NSG mice received 2.5 Gy total body irradiation and were injected with 5×10^6^ human PBMC i.v. 24h later (day 0). They were injected with either PBS (daily from day 4 to 6), 15 mg/kg of cyclosporin A (CSA, daily from day 4 to 6), 10 mg/kg of ATG (day 6) or 50.000 IU of IL-2 (24h, 12h and 1h before sacrifice). At day 7, animals were sacrificed and spleens were collected for flow cytometry comparison of CD4^+^CD25^high^FOXP3^+^ cells frequency **A.** and _phospho_STAT5 level in T_regs_ **B.** between the different treatments (PFE technique was used for permeabilization). Data show median values of 8 biological replicates with interquartile range (* *p* < 0.05, **p < 0.005, ***p < 0.0005).

Immunosuppressive drugs, such as anti-thymocyte globulin (ATG) and cyclosporin A (CSA) are widely used to prevent or treat pathologies where T_convs_ and T_regs_ play a critical role such as GVHD [26]. To gain further insight on the impact of these drugs on T_regs_, we assessed their impact on _phospho_STAT5 levels using the PFE technique. We first assessed this impact *in vitro* and observed that ATG dramatically increased the frequency of CD4^+^CD25^high^FOXP3^+^ T_regs_ (as observed previously by other investigators [27, 28]), while T_reg_ frequency was reduced by CSA (Figure 3A). Interestingly, both ATG and CSA significantly reduced _phospho_STAT5 levels in T_regs_ (Figure 3B). This suggests that T_reg_ expansion by ATG is independent of STAT5 signaling and is rather the result of the conversion of T_convs_ into T_regs_, as previously showed by other investigators [27]. In addition, the lowered levels of _phospho_STAT5 by CSA are probably the results of its inhibitory effect on IL-2 production.

We next investigated the impact of these compounds *in vivo* in NSG mice transplanted with human PBMCs. We observed that, in agreement with *in vitro* experiments, ATG dramatically increased T_reg_ frequency, while the opposite was observed with CSA (Figure 3C). Further, while IL-2 significantly increased _phospho_STAT5 levels in T_regs_, the opposite was observed with ATG and CSA (Figure 3D).

### Validation of a multicolor staining to monitor _phospho_STAT5 levels in mouse T_reg_

**Figure 4:**
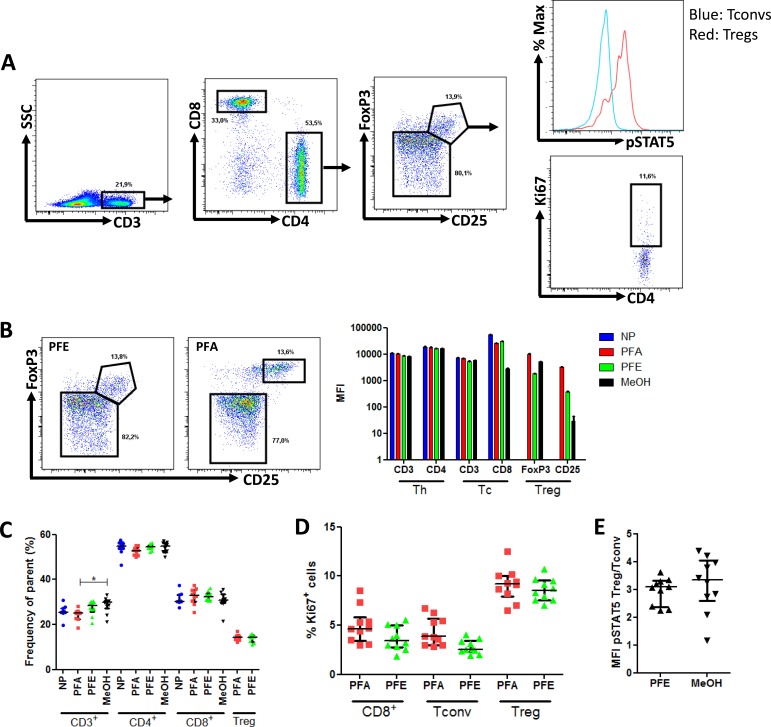
Phospho-STAT5 analyzes in murine samples Splenocytes from 10 mice were surface stained with anti-CD3, -CD4, -CD8 and -CD25 antibodies and were either non permeabilized (NP) or stained with anti-FoxP3, -Ki67 and -_phospho_STAT5 antibodies after permeabilization with either paraformaldehyde (PFA)-based technique, PerFix EXPOSE (PFE) or methanol (MeOH)-based technique. Data show median values of 10 biological replicates / condition with interquartile range (* *p* < 0.05, **p < 0.005, ***p < 0.0005). **A.** Gating strategy of tubes permeabilized with the PFE technique. **B.** Impact of PerFix permeabilization on CD25 and FoxP3 staining (left panel) and on staining of markers of helper (Th), cytotoxic (Tc) and regulatory (T_reg_) T cells (right panel) **C.** Comparison of cell frequencies between the different permeabilization techniques: CD3^+^ T cells among total splenocytes, CD4^+^ helper and CD8^+^ cytotoxic cells among CD3^+^ T cells and CD25^high^FoxP3^+^ among CD4^+^ T cell. **D.** Comparison of Ki67^+^ cell frequency within CD8^+^ T cells, T_convs_ and T_regs_ between the PFA-based and PFE techniques. **E.** Comparison of ratio, for each sample, of _phospho_STAT5 MFI of T_regs_ *versus*_phospho_STAT5 MFI of T_convs_ in PerFix and methanol-based methods.

We next assessed the capacity of the PFE kit to allow the accurate quantification of _phospho_STAT5 levels in combination with surface (CD3, CD4, CD8, CD25) and non-phosphorylated intracellular (FOXP3 and Ki67) epitopes in murine splenocytes from 5 B10.D2 and 5 B10.BR mice (Figure 4A). The following combination of antibodies was used: CD3-V500, CD4-eFluor450, CD8-PE-Cy7, CD25-PerCP-Cy5.5, FoxP3-PE, Ki67-FITC and _phospho_STAT5-AlexaFluor647 (detailed in material and methods).

In slight contrast to what was observed with human PBMCs, the PFE kit slightly reduced the expression (MFI) of both CD25 and FoxP3 by T_regs_, while no impact was observed on CD3, CD4 and CD8 staining (Figure 4B). However, importantly, the frequencies of CD3^+^, CD3^+^CD4^+^, CD3^+^CD8^+^, and CD3^+^CD4^+^CD25^+^FoxP3^+^ lymphocytes were similar between PFE and either not permeabilized or PFA samples (Figure 4C). Further, the percentage of cells expressing the proliferation marker Ki67 was also comparable with the PFA and PFE kits (Figure 4D). In contrast, methanol-based permeabilization resulted in an almost complete loss of CD25 staining hampering proper gating of CD3^+^CD4^+^CD25^+^FoxP3^+^ cells.

Next, we compared the T_reg_/T_conv_ ratio of _phospho_STAT5 levels between PFE and methanol-based permeabilization and found no significant differences (Figure 4E).

Finally, we investigated whether the alteration of CD25 and FoxP3 staining with PFE could be reproduced with different antibodies than CD25-PerCP-Cy5.5 and FoxP3-PE that were used here above. As shown in Supplemental Figure 4, CD25-PE showed satisfactory staining in comparison to the PFA condition while FoxP3-APC staining was completely lost. These results demonstrate that, for optimal results, the deleterious impact of PFE on specific antibodies should be evaluated before performing any analysis.

Altogether these data validate a 7-color staining method allowing measurement of _phospho_STAT5 levels together with Ki67 expression in mouse T-cell samples.

### In mouse splenocytes, naive T_regs_ have higher _phospho_STAT5 levels than effector T_regs_ after stimulation with IL-2

**Figure 5:**
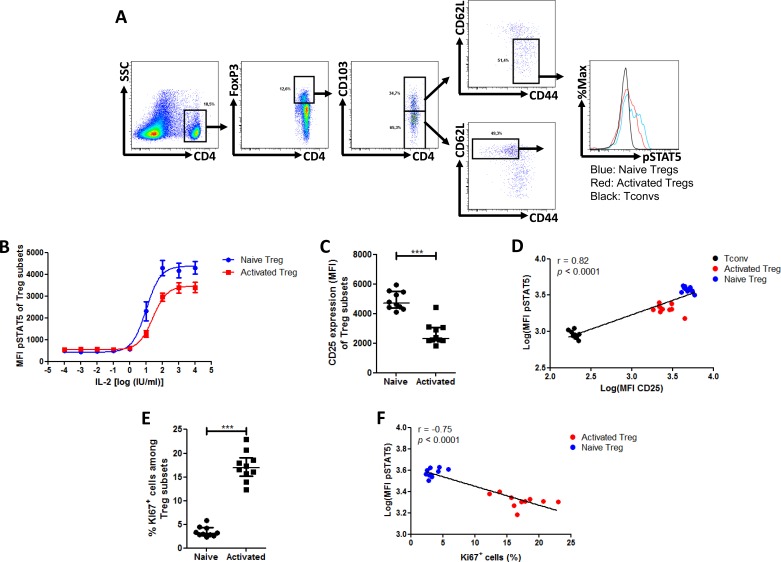
Phospho-STAT5 comparison between murine T_reg_ subsets Mouse splenocytes were isolated from 10 animals, stimulated with 10 IU/ml IL-2, stained with anti-CD4, -CD25, -CD103, -CD62L, -CD44 surface antibodies, permeabilized with the PerFix EXPOSE method and stained with anti-FoxP3, -Ki67 and -_phospho_STAT5 antibodies. Data show median values of 10 biological replicates / condition with interquartile range (* *p* < 0.05, **p < 0.005, ***p < 0.0005). **A.** Gating strategy used to compare the naive (CD4^+^FoxP3^+^CD44^low^CD62L^high^CD103^−^) and activated (CD4^+^FoxP3^+^CD44^high^CD62L^low^CD103^+^) T_reg_ subsets. **B.** Splenocytes were isolated from 3 B10.D2 mice, stimulated with various concentrations of IL-2 for 15 min and _phospho_STAT5 level was measured in T_reg_ subsets after PFE permeabilization. **C.** Comparison of CD25 expression (MFI) between the different T_reg_ subsets. **D.** Correlation between STAT5 phosphorylation level (log of MFI) and CD25 expression (log of MFI) in T_convs_ and the different T_reg_ subsets after stimulation with 10 IU/ml of IL-2. **E.** Comparison of Ki67^+^ cell frequency among the different T_reg_ subsets. **F.** Correlation between STAT5 phosphorylation level (log of MFI) and Ki67^+^ cell frequency in the different T_reg_ subsets after stimulation with 10 IU/ml of IL-2.

Because IL-2 and _phospho_STAT5 play a major role in mouse T_reg_ homeostasis [29], we compared the _phospho_STAT5 response curve of naive and effector mouse T_regs_ to various concentrations of IL-2 (Figure 5A) by using the following antibodies: CD4-eFluor450, CD25-PE-Cy7, CD62L-APC-eFluor780, CD44-FITC, CD103-BV510, FoxP3-PE, Ki67-PerCP-Cy5.5 and _phospho_STAT5-AlexaFluor647 (detailed in material and methods). In contrast to what was observed in human T_regs_, murine naive T_regs_ responded to lower concentrations of IL-2 than effector T_reg_ (Figure 5B). This is illustrated by the significant difference of log(EC_50_) between the two subsets (naive T_regs_: 1.022 +/− 0.105 vs effector T_regs_: 1.416 +/− 0.088 (*p* = 0.0078)). This is consistent with a previous report showing that IL-2 injections in mice stimulated to a higher extent the proliferation of CD44^low^CD62L^high^CCR7^+^ than CD44^high^CD62L^low^CCR7^−^ T_regs_ [30, 31]. Further, we investigated the respective expression of CD25 in both subsets and found that naive T_regs_ expressed higher levels of CD25 than effector T_regs_, in agreement with their response curves to IL-2 (Figure 5C) and with recently reported data [31]. As observed in human samples and taking data from T_conv_ and T_reg_ subsets together, there was a strong correlation between _phospho_STAT5 and CD25 levels (Spearman *r* = 0.82, *p* < 0.0001, Figure 5D). However we found that activated T_regs_ expressed dramatically higher levels of Ki67 than naive T_reg_ (Figure 5E) and an inverse correlation between _phospho_STAT5 level and Ki67 was observed when taking data from T_reg_ subsets together (Spearman *r* = −0.75, *p* < 0.0001, Figure 5F). Because the time of exposure to IL-2 in our experiments (15 min) is insufficient to induce Ki67 expression through the _phospho_STAT5 pathway (similar frequencies of Ki67 were found between stimulated and unstimulated T_regs_, data not showed), these data suggest that T_reg_ proliferation is conditioned by their activation status while their capacity to respond to IL-2 is conditioned by their expression of CD25.

In summary, we have validated a multicolor staining method that allows the measurement of STAT5 phosphorylation levels in human and murine T_reg_ subsets. Further, comparison of _phospho_STAT5 levels in response to IL-2 between the different T_reg_ subsets highlighted a striking divergence between the two species. These observations are in line with recent reports showing higher CD25 expression in activated effector T_regs_ than in naive T_regs_ in human PMBCs [8], but higher CD25 expression in naive T_regs_ than in effector T_regs_ in mouse splenocytes [31]. Further, as IL-2 administration has been previously shown to increase naive T_reg_ frequency in mouse [31] and activated ICOS^+^ T_reg_ in humans [25], our work suggest that *ex vivo* response curves to cytokines might be predictive of the impact of specific cytokine administration on the homeostasis T_regs_ and other T-cell population.

## MATERIALS AND METHODS

### Volunteers

Blood from healthy adult volunteers (aged 21 to 37 years) was collected following written informed consent (approved by our local Ethic Committee). Peripheral blood mononuclear cells (PBMCs) were immediately isolated by density gradient centrifugation (Ficoll-Paque™, GE Healthcare, Upsala, Sweden) and then resuspended in staining buffer PBS+3%FBS (Lonza, Verviers, Belgium) at a concentration of 1×10^6^/ml.

### Animal experiments

For the study assessing the impact of immunosuppressive drugs on human cells *in vivo*, NOD-scid IL-2Rγ^null^ (NSG) (The Jackson laboratory, Bar Harbor, ME) mice, aged from 8 to 9 weeks, were irradiated (2.5 Gy total body irradiation) and transplanted i.v. 24h later with 5×10^6^ human PBMCs (freshly isolated from one healthy volunteer). Animal weight and survival were monitored daily, in agreement with the recommendation of our ethical committee. Mice were then injected intraperitoneally with either PBS daily on days 4, 5 and 6 post-transplantation, 15 mg/kg of cyclosporin A (CSA, Sandimmune, Novartis, Basel, Switzerland) daily on days 4, 5 and 6 post-transplantation, 10 mg/kg of ATG (ATG-Fresenius, Neovii Biotech GmbH, Gräfelfing, Germany) on day 6 post-transplantation, or 50.000 IU of IL-2 (Proleukin, Novartis), 25h, 15h and 1h before the sacrifice at day 7.

For murine T_reg_ and _phospho_STAT5 analyses, mice of either the B10.BR or B10.D2 strains (aged from 8-10 weeks) were sacrificed according to the local ethics policies without receiving any particular treatment. Spleens of either NSG, B10.BR or B10.D2 mice were collected in RPMI supplemented with 5% FBS and were prepared to a single cell suspension. Cells were washed twice in staining buffer, filtered through a 75 μm nylon mesh to remove aggregates, and were resuspended at a concentration of 1×10^6^ cells/ml in staining buffer.

### Cell culture and suppression assays

For *in vitro* drugs assays, freshly isolated human PBMCs from 3 healthy volunteers were activated in triplicate in the presence of anti-CD3/CD28 beads (Bead-Cell ratio 1:1, Invitrogen, Waltham, MA) in complete medium that consisted of RPMI 1640 L-glutamine (2mM) (Lonza) supplemented with penicillin (100 IU/ml) (Lonza), streptomycin (10 mg/ml) (Lonza) and 10% human AB serum (Sigma-Aldrich, St. Louis, MO). Culture medium was supplemented with either PBS, 500 ng/ml of CSA, or 100 μg/ml of ATG Fresenius/Neovii. Cells were collected for flow cytometry analysis after 24h of incubation at 37°C, 5% CO_2_.

For suppression assays, T_reg_ subsets were isolated from human PBMCs by flow cytometry (FACS Aria III, Becton Dickinson (BD), Bedford, MA). Sorted naive T_convs_ were loaded with CFSE 5 μM (Invitrogen) and used as responder cells. Assays were performed in complete medium in the presence of anti-CD3/CD28 beads (Bead-Cell ratio 1:8, Invitrogen). Sorted T_regs_ and responder cells were cultured in mixed lymphocyte reactions at different T_reg_:T_resp_ ratios for 72h before analysis by flow cytometry.

### IL-2 stimulation

Human PBMCs or murine splenocytes (1×10^6^ cells/ml in staining buffer) were stimulated with human recombinant IL-2 (Peprotech EC ltd., London, UK) because recombinant human IL-2 has been proven to be also efficient in mouse [32, 33]. For all experiments other than dose-response curves, a concentration of 10 IU/ml was used, while for dose-response curve experiments, an 8-log range of concentrations was used for human and a 9-log range was used for murine experiments. Samples were stimulated for 15 min at 37°C and were immediately washed twice with staining buffer before processing for flow cytometry staining. This time of exposure was chosen based on _phospho_STAT5 levels observed in lymphocytes after different times of stimulation with 10 IU/ml (Supplemental Figure 5), 15 min corresponding to a time point of maximal response.

### Flow cytometry

The following antibodies specific for human epitopes were used: CD4-PE-Cy5 (RPA-T4), CD25-BV421 (M-A251, BD), CD127-biotin (eBioRDR5), CD45RA-BV510 (HI100, BD), HLA-DR-APC-efluor780 (LN3), FOXP3-AlexaFluor488 (259D, Biolegend, ImTech Antwerp, Belgium), posphoSTAT5-AlexaFluor647 (p7694, BD), Ki67-FITC (B56, BD), KI67-PerCP-Cy5.5 (B56, BD), KI67-PE (B56, BD) and anti-streptavidin-PE-Cy7. The following antibodies specific for mouse epitopes were used: CD3-V500 (500A2, BD), CD4-eFluor450 (RM4-5), CD8-PE-Cy7 (53-6.7), CD25-PerCP-Cy5.5 (PC61.5), CD25-PE-Cy7 (PC61, BD), CD25-PE (PC61.5), CD103-BV510 (M290, BD), CD44-FITC (IM7), CD62L-APC-eFluor780 (MEL-14), FoxP3-APC (FJK-16s), and FoxP3-PE (FJK-16s) (all from eBioscience, unless indicated otherwise). Cells (1×10^6^ cells/sample) were incubated with surface antibodies for 20 min at 4°C in the dark and washed with staining buffer. This process was repeated for a 15-minute period for the streptavidin staining step. Then, samples were either not permeabilized and kept in PBS at 4°C until analysis by flow cytometry (Non Permeabilized (NP) condition) or were permeabilized by the use of one of three different procedures: the paraformaldehyde (PFA)-based method with FOXP3 Staining Buffer Set (eBioscience); the methanol (MeOH)-based method with Lyse/Fix buffer (Becton Dickinson) followed by Phosflow Perm Buffer III (Becton Dickinson) (Methanol-based method) or the PerFix EXPOSE (PFE, Beckman Coulter, Brea, CA) method. Manufacturer instructions were followed for each procedure. Data were acquired on a FACS Canto II (Becton Dickinson) and were analyzed with FlowJo v7.6.5 (Treestar Inc., San Carlos, CA).

### Gating strategy and definition of T_reg_ subsets

In human samples, T_regs_ were defined as CD4^+^CD25^+^CD127^dim^FOXP3^+^ lymphocytes. Naive T_regs_ were defined as CD45RA^+^HLA-DR^neg^ T_regs_, activated effector T_regs_ were defined as CD45RA^neg^HLA-DR^+^ T_regs_, while HLA-DR^neg^ effector T_regs_ were defined as CD45RA^neg^HLA-DR^neg^ T_regs_ as recently proposed by Dong *et al.* [9]. In murine samples, T_regs_ were defined as CD4^+^CD25^+^FoxP3^+^ lymphocytes. Naive T_regs_ were defined as CD4^+^FoxP3^+^CD44^low^CD62L^high^CD103^−^ lymphocytes while effector T_regs_ were defined as CD4^+^FoxP3^+^CD44^high^CD62L^low^CD103^+^ lymphocytes [4].

### Statistical analyses

The Mann-Whitney *U* test was used to compare flow cytometry data from different treatments. The extra sum-of-squares *F* test was used to compare the half maximal effective concentrations (EC_50_). The Spearman test was used to assess correlations. Statistical analyses were carried out with Graphpad Prism 5.0 (Graphpad Software, San Diego, CA). *P* values < 0.05 were considered as statistically significant.

## SUPPLEMENTARY MATERIAL AND FIGURES


