# Supplementary material for: Validation of a multicolor staining to monitor phosphoSTAT5 levels in regulatory T-cell subsets
Source: Oncotarget. 2015 Dec 7;6(41):43255–66. doi: 10.18632/oncotarget.6486 (PMC4791230; doi:10.18632/oncotarget.6486)
Supplement: Supplementary file 1 [file oncotarget-06-43255-s001.pdf]

Validation of a multicolor staining to monitor $_{phospho}$STAT5 levels in regulatory T-cell subsets

Supplementary Material

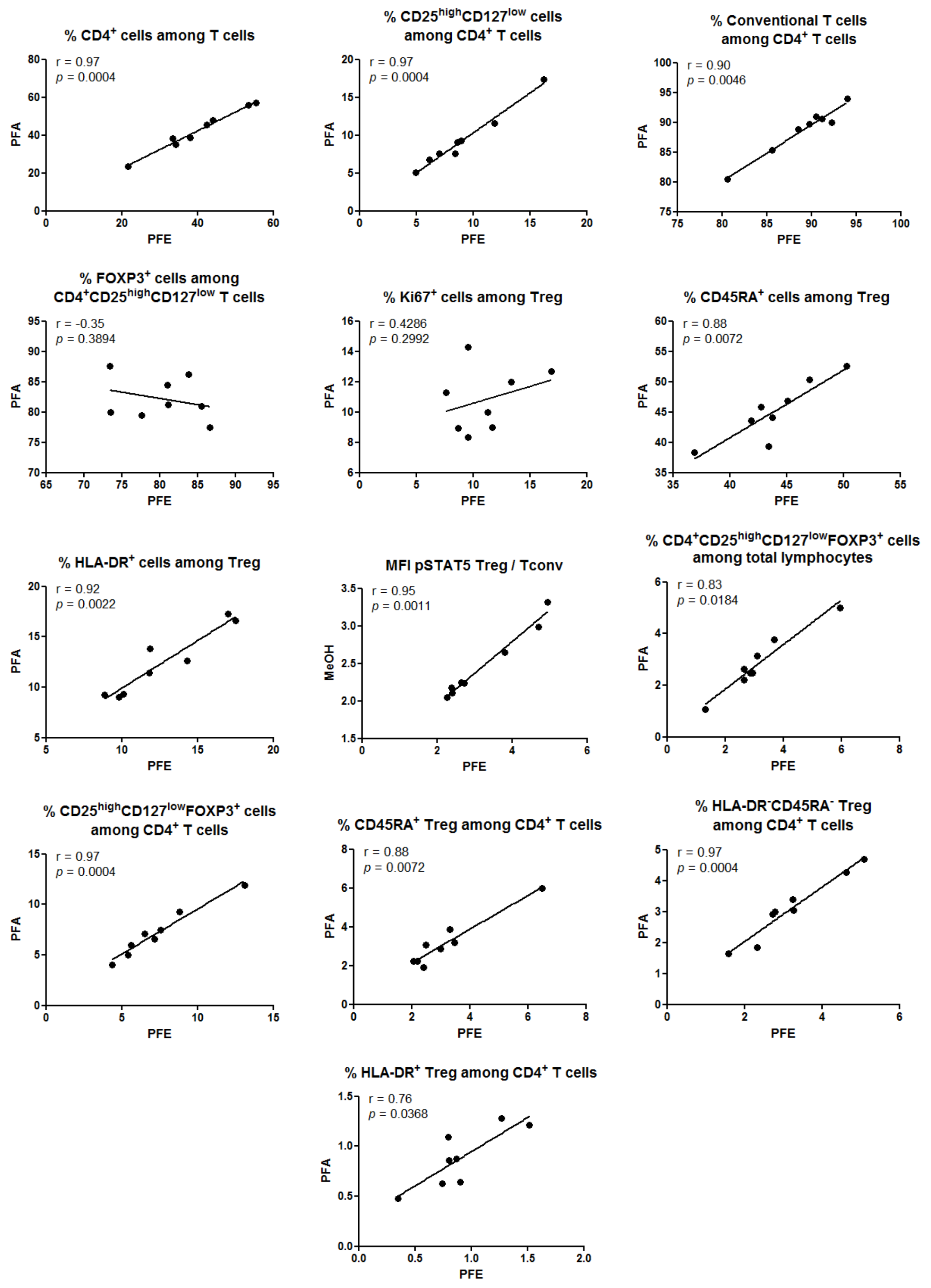

Supplemental Figure 1: PBMC from 8 healthy volunteers were surface stained with anti-CD4, -CD25, -CD127, -CD45RA and -HLA-DR antibodies and were stained with anti-FOXP3, -KI67 and -$_{phospho}$STAT5 antibodies after permeabilization with either paraformaldehyde (PFA), PerFix EXPOSE (PFE), or methanol (MeOH)-based technique. Results for the different analyzed parameters were correlated (spearman correlation) between the PFE and reference method (PFA for extracellular and non-phosphorylated intracellular epitopes or MeOH for phosphorylated epitopes ($_{phospho}$STAT5)), for each donor.

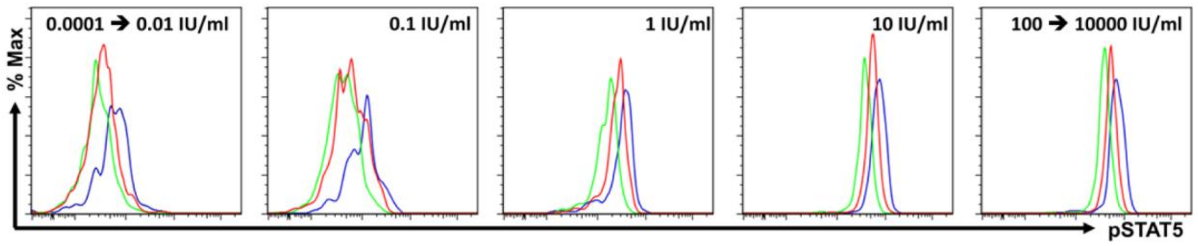

Supplemental Figure 2: Representative histograms of dose-response curve experiment comparing human T_{reg} subset phosphorylation level of STAT5 after stimulation with increasing doses of human recombinant IL-2.

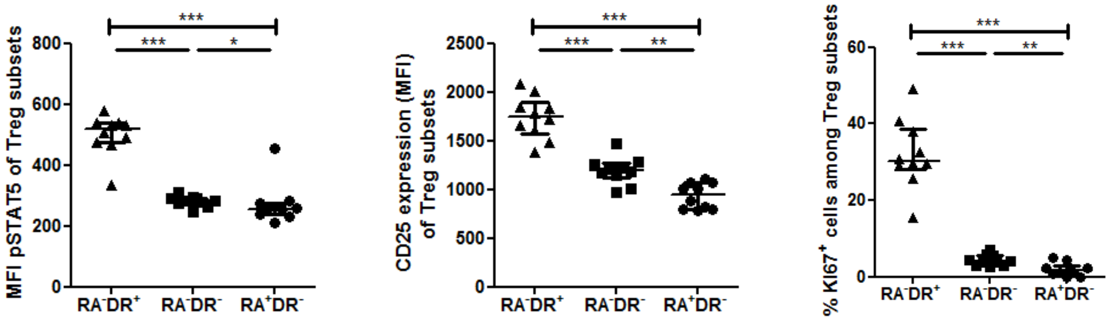

Supplemental Figure 3: PBMCs from 8 healthy volunteers were cryopreserved, thawed one week later, surface stained with anti-CD4, -CD25, -CD127, -CD45RA and -HLA-DR antibodies and were stained with anti-FOXP3, -KI67 and -$_{phospho}$STAT5 antibodies after permeabilization with PerFix EXPOSE (PFE) technique. $_{Phospho}$STAT5, CD25 and KI67 expression were then compared between the different Treg subsets. Data show median values of 8 biological replicates / condition with interquartile range (* *p*< 0.05, ***p*< 0.005, ****p*< 0.0005).

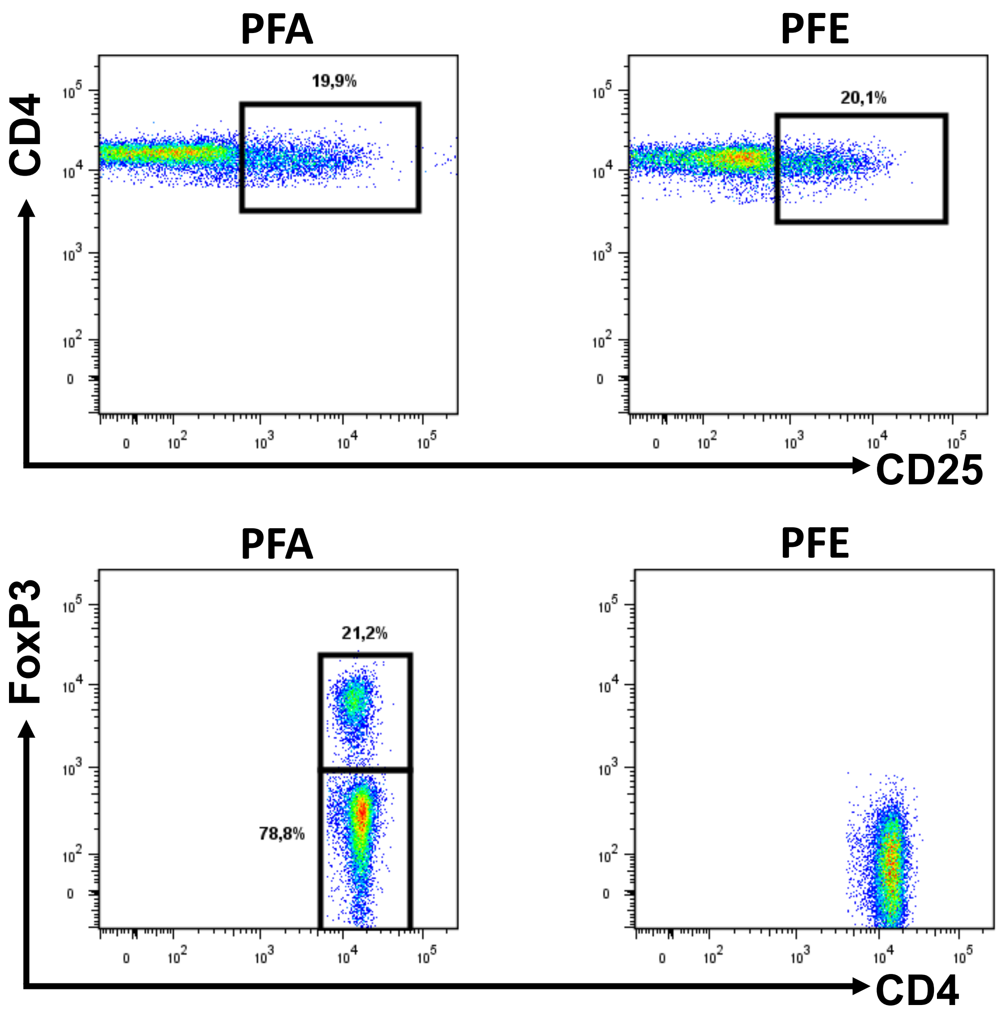

Supplemental Figure 4: Comparison of anti-mouse CD25-PE and FoxP3-APC antibody staining between paraformaldehyde (PFA)-based and PerFix EXPOSE (PFE) permeabilizations.

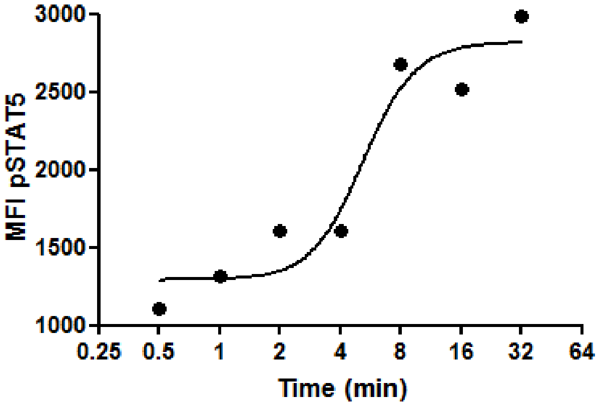

Supplemental Figure 5: PBMCs (1x10^6 freshly isolated cells) from one healthy volunteer were exposed to 10 IU/ml of IL-2 for different time periods and were stained with anti-CD4, -CD25 and -CD127 antibodies and were stained with anti-FOXP3, and -$_{phospho}$STAT5 antibodies after permeabilization with PerFix EXPOSE (PFE) technique. $_{Phospho}$STAT5 level in Treg was then measured and plotted versus time of exposure.
